# Novel roles of sulfur metabolism in stress‐controlled stomata aperture regulation

**DOI:** 10.1111/nph.71048

**Published:** 2026-03-02

**Authors:** Sheng‐Kai Sun, Rüdiger Hell, Markus Wirtz

**Affiliations:** ^1^ Centre for Organismal Studies (COS) Heidelberg University Heidelberg 69120 Germany

**Keywords:** 3′‐phosphoadenosine 5′‐phosphate, ABA biosynthesis, cysteine biosynthesis, cysteine synthase complex, guard cells, stomata closure, sulfate, sulfide, sulfur

## Abstract

Stomatal closure allows plants to conserve water by reducing transpiration during drought. Surprisingly, the assimilation of the macronutrient sulfur is intimately connected to the drought stress response. This Tansley insight will only briefly touch on the general impact of sulfate assimilation on the production of drought‐response metabolites. Instead, the emphasis will be on the unexpected role of cysteine in triggering guard cell‐autonomous abscisic acid biosynthesis in response to diverse drought‐associated stresses. A particular focus will be on identifying the chloroplast‐localized cysteine synthase complex as a sensor hub that integrates long‐distance soil‐drying signals and local high‐light signals to mediate stress‐induced stomatal closure. Furthermore, we will discuss the emerging role of cysteine‐derived sulfide as a signal in stomatal closure.

**Table:** 

	Content	
	Summary	1460
I.	Introduction	1460
II.	The role of the cysteine synthase complex in primary sulfur metabolism	1462
III.	Sulfate acts as a soil‐borne signal for stomata closure	1462
IV.	Cytosolic sulfide controls various stress responses, including stomata closure	1465
V.	Conclusions and outlook	1465
	Acknowledgements	1465
	References	1466

## I. Introduction

**Fig. 1:**
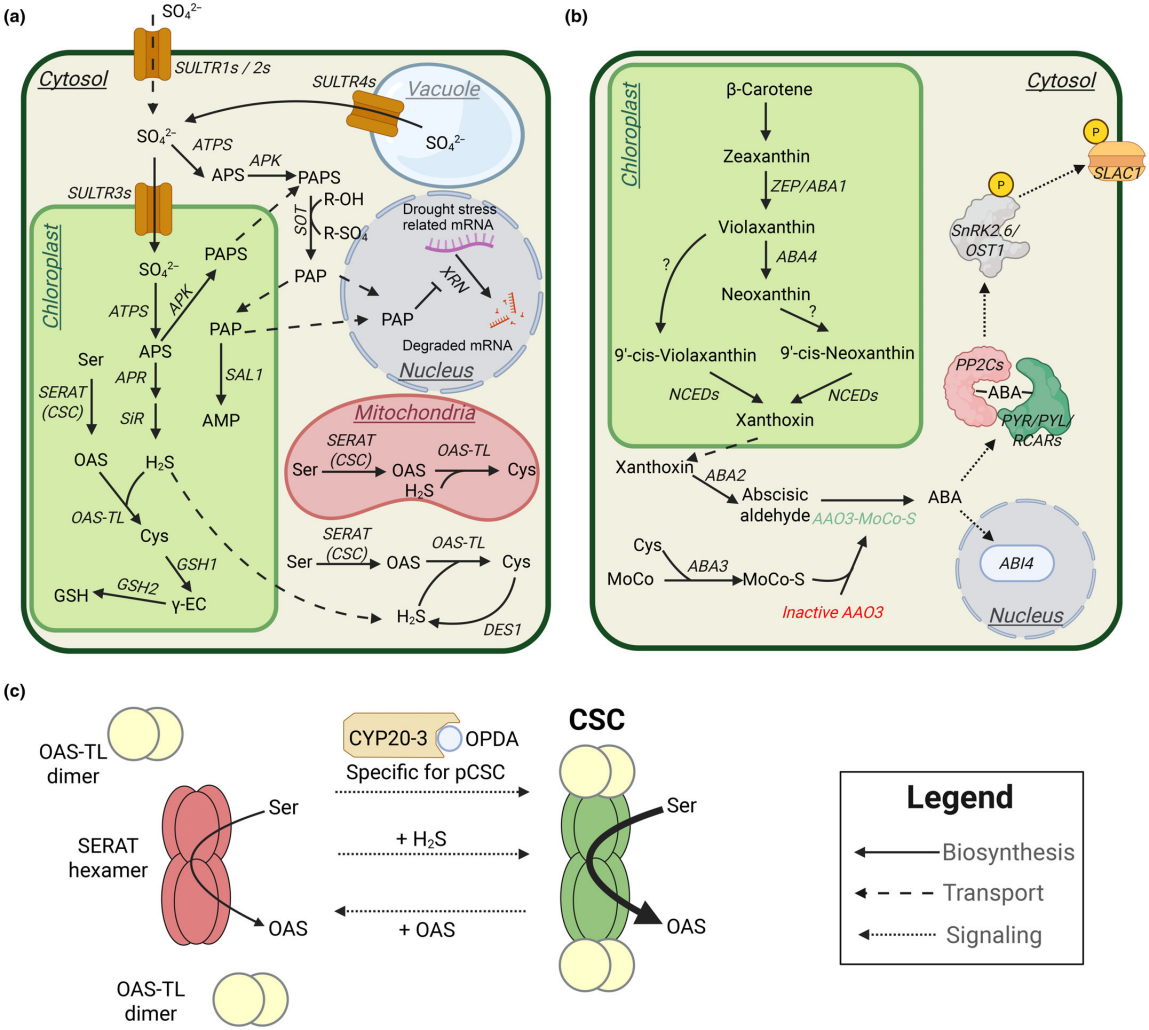
Schematic overviews of the sulfur assimilation pathway, the abscisic acid signaling pathway, and the regulatory function of the cysteine synthase complex. (a, b) Subcellular localization of the sulfur metabolism pathway (a) and the biosynthesis and perception of ABA (b) in plant cells. Enzymes and transporters are indicated in italics. Metabolites are shown in regular font. Solid black lines indicate chemical reactions catalyzed by enzymes. Transport of metabolites is represented in dashed black lines. Dotted lines indicate the impact of signaling molecules on enzymes or metabolism. (c) Model for regulation of cysteine synthesis by the cysteine synthase complex. The cysteine synthesis limiting enzyme SERAT (red, inactive) is activated by the formation of the CSC (green, active). OAS‐TL acts within the CSC as a regulatory subunit that controls SERAT activity. For a detailed description of the action of the effectors, see the main text. Metabolite abbreviations: ABA, abscisic acid; AMP, adenosine monophosphate; APS, adenosine 5′‐phosphosulfate; Cys, cysteine; γ‐EC, γ‐glutamylcysteine; GSH, glutathione; MoCo, molybdenum cofactor; MoCo‐S, sulfurylated molybdenum cofactor; OAS, *O*‐acetylserine; OPDA, 12‐oxo‐phytodienoic acid; PAPS, 3′‐phosphoadenosine 5′‐phosphosulfate; PAP, 3′‐phosphoadenosine 5′‐phosphate; S^2−^, sulfide, Ser, serine. SO_4_^2−^, sulfate. Protein abbreviations: AAO3, aldehyde oxidase 3; ABA2, Short‐chain dehydrogenase/reductase ABA DEFICIENT 2; ABA3, sulfurtransferase ABA DEFICIENT 3; ABA4, ABA DEFICIENT 4; ABI4, ABA INSENSITIVE 4; APK, APS kinase; APR, APS reductase; ATPS, ATP sulfurylase; CYP20‐3, cyclophilin 20–3; DES1, L‐cysteine desulfhydrase 1; GSH1, γ‐glutamylcysteine synthetase; GSH2, glutathione synthetase; NCED, Nine‐*cis*‐epoxycarotenoid dioxygenase; OAS‐TL, *O*‐acetylserine(thiol)lyase; PP2C, Type 2C protein phosphatase; PYR/PYL/RCAR, pyrabactin resistance/PYR1‐like/regulatory components of ABA receptor; SAL1, nucleotide phosphatase; SERAT, serine acetyltransferase; SiR, sulfite reductase; SLAC1, Slow anion channel‐associated 1; SnRK, Snf1‐related protein kinase; SOT, sulfotransferase; SULTR, sulfate transporter; XRN, 5′ to 3′‐exoribonuclease; ZEP/ABA1, Zeaxanthin epoxidase ABA DEFICIENT 1. This figure was created in BioRender (BioRender.com/4vg6oit).

Rapid changes in environmental cues force plants to dynamically adapt their metabolism and growth. Research over the last decades has shown that sulfur metabolism is intrinsically connected to drought and other stress responses by affecting hormone signaling pathways (Fabregas *et al*., 2025). For example, sulfur metabolism could crosstalk with cytokinin or brassinosteroid signaling pathway (Pavlů *et al*., 2022; Wang *et al*., 2023, 2024). The link between sulfur metabolism and the response to drought stress is so far most established (reviewed in Chan *et al*., 2013; Fabregas *et al*., 2025). Early work suggested that this connection was mainly driven by sulfur‐containing defense compounds during drought, such as the osmoprotectant choline‐O‐sulfate and the reactive oxygen species (ROS) scavenger glutathione (GSH; Fig. 1a; Hanson *et al*., 1994; Chen *et al*., 2012). Later studies, however, revealed that intermediates and by‐products of sulfur metabolism also have signaling functions within the drought stress response (Malcheska *et al*., 2017; Pornsiriwong *et al*., 2017). These signals can be either dependent on or independent of abscisic acid (ABA; Estavillo *et al*., 2011; Batool *et al*., 2018), a phytohormone controlling most aspects of the drought response (Fig. 1b), including stomatal closure (reviewed in Kuromori *et al*., 2018). One example of how sulfur metabolism‐generated signals interfere with the drought response in an ABA‐independent manner is the stress‐induced accumulation of 3′‐phosphoadenosine 5′‐phosphate (PAP). PAP is the by‐product of sulfation reactions catalyzed by sulfotransferases using PAPS as substrate (Mugford *et al*., 2009; Fig. 1a). Upon drought stress, PAP accumulates and inhibits the RNA‐degrading activity of 5′‐3′‐exoribonucleases, which control the accumulation of drought stress‐associated transcripts in the cytosol (Estavillo *et al*., 2011). PAP accumulation depends on the redox‐sensitive enzyme SAL1. As SAL1 is located in chloroplasts and mitochondria, PAP is supposed to serve as a retrograde drought stress signal that can trigger stomatal closure (reviewed in Chan *et al*., 2013; Pornsiriwong *et al*., 2017). However, in this Insight, we focus on primary sulfur metabolism‐related signals controlling stomatal closure in an ABA‐dependent manner.

## II. The role of the cysteine synthase complex in primary sulfur metabolism

Primary sulfur metabolism starts with the uptake of sulfate from the soil by sulfate transporters of Group 1 (SULTR1s). The symplastic sulfate is transported via the xylem to the leaves for assimilatory reduction (Fig. 1a). Low‐affinity SULTRs of Group 2 import sulfate into the leaf cells, where it is stored in the vacuole or transported into the chloroplast by SULTRs belonging to Group 3 (Chen *et al*., 2019). In the chloroplast, the sulfate is activated by ATP sulfurylase and reduced to sulfide by adenosine 5′‐phosphosulfate (APS) reductase and sulfite reductase. Sulfide can be incorporated into cysteine by *O*‐acetylserine(thiol)lyase (OAS‐TL) either in the chloroplast and the cytosol or in the mitochondria. To this end, serine acetyltransferase (SERAT) produces the *O*‐acetylserine (OAS), which accepts the sulfide. SERAT and OAS‐TL interact reversibly in the hetero‐oligomeric cysteine synthase complex (CSC), resulting in substantial activation of SERAT. Sulfide promotes CSC formation, whereas OAS causes its dissociation, allowing cysteine synthesis to be regulated according to the availability of sulfur and carbon/nitrogen precursors (Fig. 1c). Remarkably, the CSC is present in all subcellular compartments capable of protein biosynthesis (Wirtz & Hell, 2006). In the following chapters, we outline how drought stress‐induced alterations in sulfur metabolism are transduced into rapid changes in ABA synthesis and how CSCs contribute to these dynamic adaptations.

## III. Sulfate acts as a soil‐borne signal for stomata closure

**Fig. 2:**
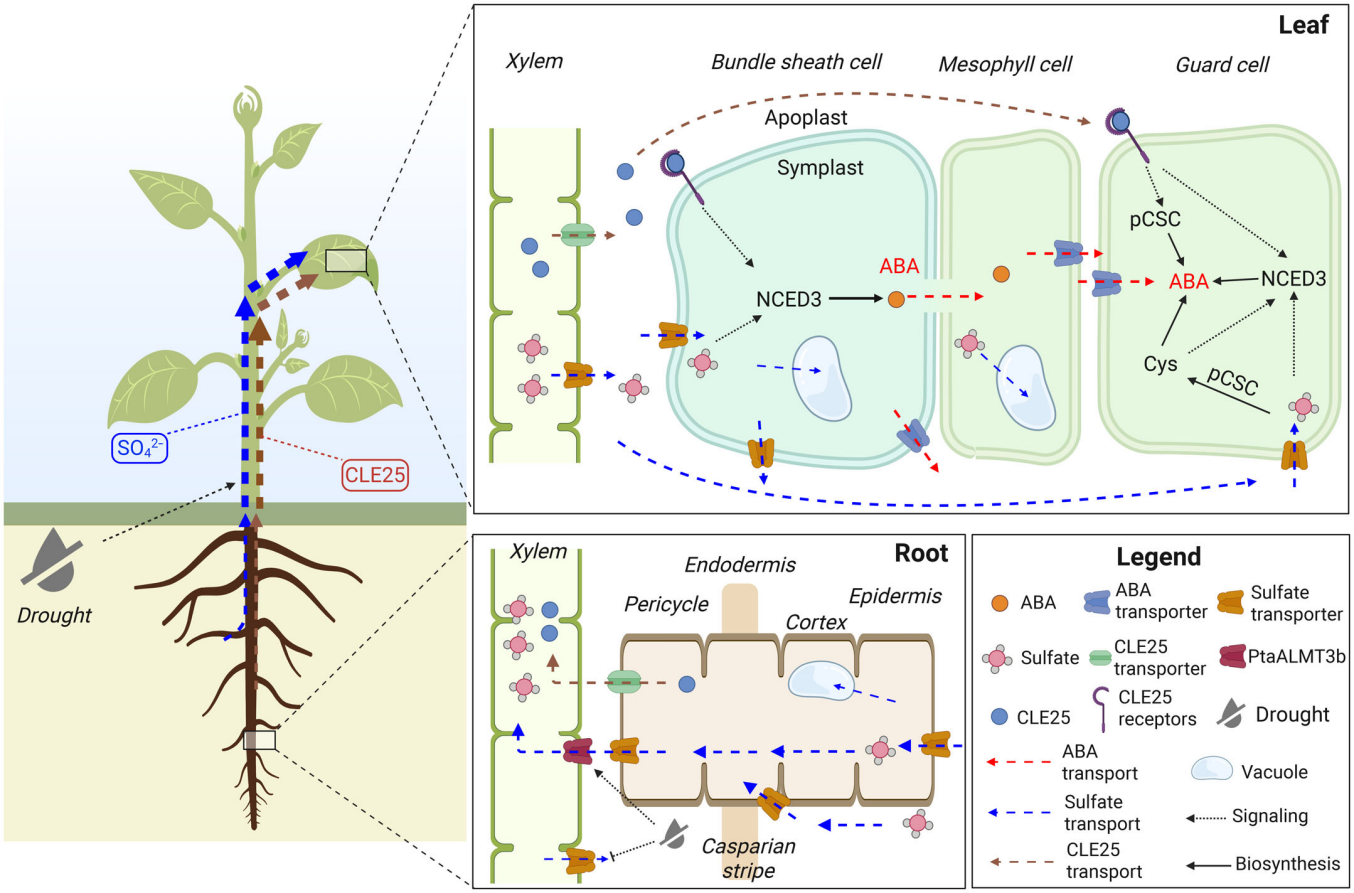
Overview of long‐distance soil‐drying signaling. Schematic depiction of long‐distance signal transport and local production of signals in response to drought‐induced soil drying. Soil drying causes and triggers transport of sulfate and the peptide‐hormone CLAVATA3/EMBRYO‐SURROUNDING REGION RELATED 25 (CLE25) from the roots to the shoots. In the leaves, both signals are unloaded into the apoplast and transported to the guard cells, which are not connected to the symplast of mesophyll or pavement cells via plasmodesmata. In case of sulfate, drought stress is supposed to trigger xylem‐loading via the anion transporter ALMT3b and decreased Xylem unloading via sulfate transporters (SULTRs) in roots (Malcheska *et al*., 2017). The mechanism by which soil drying induces the transport of CLE25 from the roots via the Xylem to the shoot remains unknown. This figure was created in BioRender (BioRender.com/bo2d4v9).

Drought stress is a combination of decreased air humidity and soil water limitation, and is often associated with elevated temperatures and high‐light stress. While decreased air humidity, elevated temperature, and intense light also promote water deficit, the most critical parameter in drought perception is the sensing of soil water availability. Grafting experiments proved that root‐derived ABA is dispensable as a primary signal for soil water limitation (Christmann *et al*., 2007). Instead, sulfate was the only soil‐borne signal in the xylem sap that preceded stomatal closure and ABA biosynthesis in maize and poplar during drought (Ernst *et al*., 2010; Malcheska *et al*., 2017). In poplar, the drought‐induced increase of sulfate in the xylem sap was caused by: enhanced loading of sulfate from parenchyma cells into the xylem by the anion channel ALMT3b; and decreased xylem unloading by sulfate transporters in roots (Fig. 2; Malcheska *et al*., 2017). How plants sense declining water availability and transduce this signal into elevated sulfate levels in the xylem needs further investigation. Application of a physiologically relevant sulfate concentration via the petiole to leaves of Arabidopsis was sufficient to induce expression of 9‐*CIS*‐EPOXYCAROTENOID DIOXYGENASE 3 (NCED3), a key enzyme in ABA biosynthesis. This treatment led to increased ABA levels in the cytosol of guard cells and ultimately caused stomatal closure (Malcheska *et al*., 2017; Batool *et al*., 2018). However, it was unclear how sulfate is sensed and in which cell type sulfate induces ABA production for stomatal closure.

**Fig. 3:**
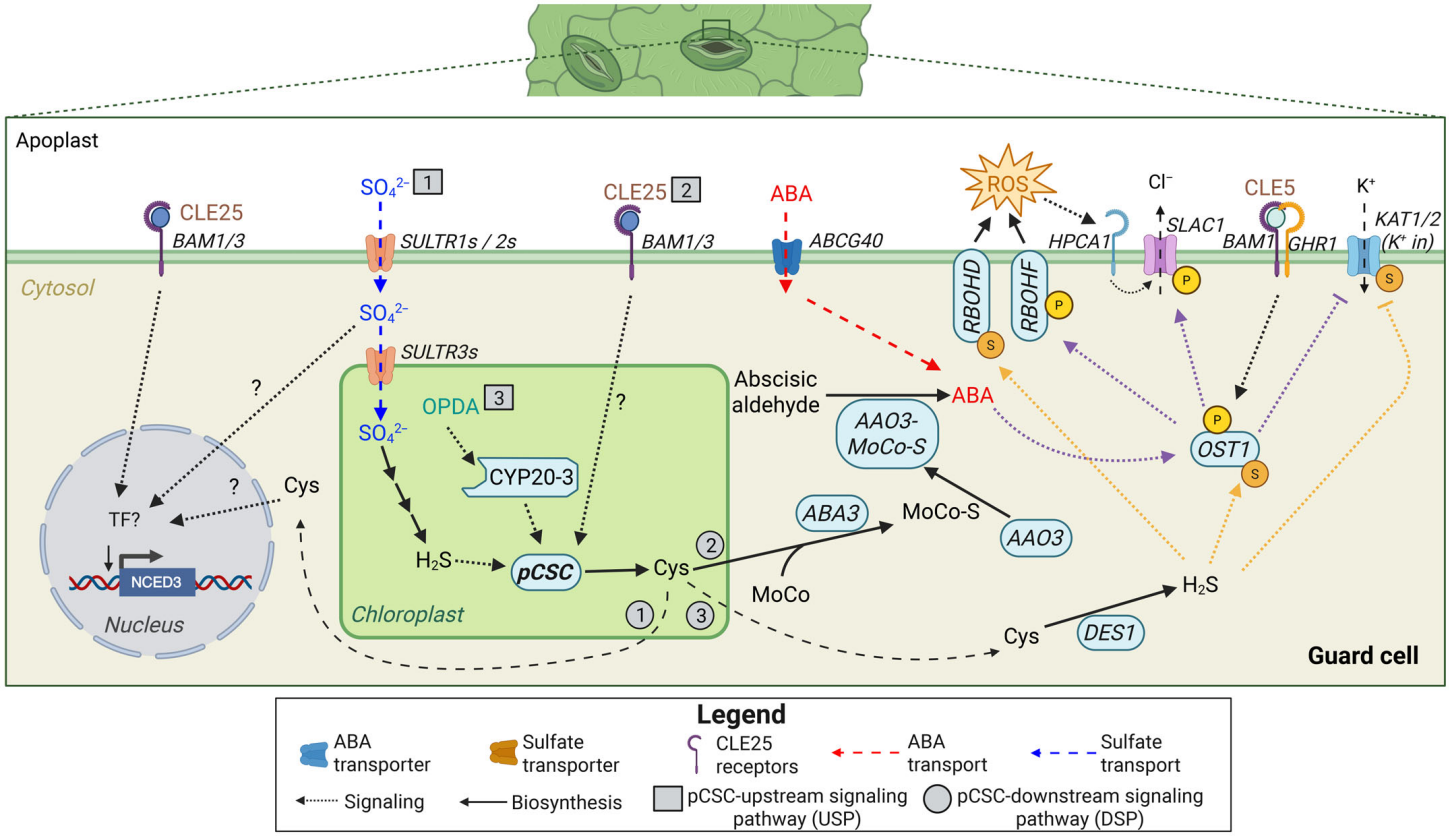
Localization of stomatal closure induction mechanisms in guard cells. Soil drying enhances the transport of sulfate and CLAVATA3/EMBRYO‐SURROUNDING REGION RELATED 25 (CLE25) from the root to the shoot, thereby increasing abscisic acid (ABA) synthesis. Sulfate‐induced ABA formation occurs at least partially, if not entirely, in the guard cells, which are capable of reducing the sulfate to sulfide for the promotion of chloroplast‐localized CSC (pCSC) formation and cysteine synthesis (upstream signaling pathway 1, USP1). Stomatal closure by CLE25 also depends on the pCSC (USP2), but the mechanistic link between CLE25 and the pCSC remains unresolved. pCSC formation‐induced cysteine can trigger stomatal closure by enhancing 9‐*CIS*‐EPOXYCAROTENOID DIOXYGENASE 3 (NCED3) transcription (downstream signaling pathway 1, DSP1) or by serving as a substrate for sulfurylation of the molybdenum cofactor (MoCO) factor by ABA‐DEFICIENT3 (ABA3), thereby activating ABSCISIC ALDEHYDE OXIDASE 3 (AAO3) (DSP2). Both pathways result in guard cell autonomous production of ABA, triggering stomatal closure. Targets of ABA signalling are indicated by purple dotted lines (arrows indicate stimulation; blunt‐ended lines indicate inhibition). Cysteine serves as the substrate of the cytosolic cysteine desulfhydrase DES1, which releases sulfide. Enhanced sulfide levels can trigger persulfidation of diverse components of the ABA signal transduction pathway, promoting stomatal closure (DSP3; yellow dotted lines; arrows indicate stimulation; blunt‐ended arrows indicate inhibition). The pCSC is also critical for high‐light stress‐induced stomata closure, which is triggered by the oxylipin signal OPDA. OPDA binds to CYP20‐3, which promotes pCSC formation (USP3). These mechanisms will ensure rapid stomatal closure upon soil drying or high‐light stress. Sustained stomatal closure causes CO_2_ limitation in leaf tissues, thereby creating signals for stomatal opening. On top of these pCSC or cysteine‐dependent mechanisms for stress‐induced ABA accumulation in guard cells, guard cells take up ABA from the apoplast via the ABCG40 transporter. This mechanism allows guard cells to sense drought‐induced ABA formation in other cells, which is particularly important during the later stages of drought stress, helping to counterbalance CO_2_ limitation‐induced stomata opening. Thus, guard cell ABA import is critical for a successful drought response, as supported by the drought‐sensitive phenotypes of many ABA transporter mutants. This figure was created in BioRender (BioRender.com/a0gklt7).

Since sulfate‐induced stomata closure was shown to require sulfate import into the chloroplasts, reduction of sulfate to sulfide, and production of OAS by SERATs, it became evident that cysteine formation is essential for sulfate‐triggered ABA production (Batool *et al*., 2018; Chen *et al*., 2019). Only the chloroplast‐localized CSC (pCSC) is necessary for sulfate‐induced stomatal closure, while the CSCs in the cytosol and mitochondria are dispensable (Sun *et al*., 2025). The pCSC not only senses the availability of sulfur (see Section I; Fig. 1c). It is also critical for the induction of stomatal closure by the peptide‐hormone CLAVATA3/EMBRYO‐SURROUNDING REGION RELATED 25 (CLE25), which independently acts as a root‐to‐shoot signal in response to soil drying (Fig. 2; Takahashi *et al*., 2018). Direct evidence for the relevance of pCSC formation in promoting ABA synthesis is provided by genetic engineering of a constitutively forming pCSC that is insensitive to OAS dissociation (Sun *et al*., 2021). *In planta*, expression of this stable pCSC results in permanent stomatal closure and enhanced tolerance towards drought stress (Sun *et al*., 2025). These findings reveal that the pCSC is a critical hub for sensing long‐distance drought signals (Fig. 3), but how does the activation of the pCSC stimulate ABA biosynthesis in leaves?

Cysteine serves as the sulfur donor for sulfurylation of the molybdenum cofactor (MoCo) by the sulfurylase ABA‐DEFICIENT3 (ABA3; Bittner *et al*., 2001). Although some molybdoenzymes, such as nitrate reductase and sulfite oxidase, function without a sulfurylated MoCo, the sulfurylated form is essential for the activity of aldehyde oxidase (AO) and xanthine dehydrogenase (XDH). Among these enzymes, ABSCISIC ALDEHYDE OXIDASE 3 (AAO3) is the primary isoform responsible for converting abscisic aldehyde into ABA (Fig. 1b; reviewed in Nambara & Marion‐Poll, 2005). NCED3 catalyzes the rate‐limiting step in abscisic aldehyde formation (Endo *et al*., 2008). Since cysteine can also induce NCED3 transcription (Batool *et al*., 2018), it remains unclear whether cysteine triggers ABA production by enhancing the biosynthesis of the AAO3 substrate, abscisic aldehyde, the AAO3 enzymatic activity, or by a combination of both (Fig. 3).

During severe drought, the NCED3 protein accumulates substantially in the vascular parenchyma cells (Endo *et al*., 2008), which is considered the dominant site of ABA production in later stages of drought (reviewed in Kuromori *et al*., 2018). ABA can be transported between different organs and within leaves between different tissues by various mechanisms. Through ABCG40 activity, guard cells can also efficiently import ABA, allowing them to perceive whole‐plant water status (reviewed in Kuromori *et al*., 2018). However, rapid stomatal closure in response to low humidity requires guard cell‐autonomous ABA biosynthesis (Yoshimoto *et al*., 2002; Bauer *et al*., 2013). Consistent with this requirement, guard cell‐specific expression of the sulfurylase ABA3 is sufficient to restore stomatal closure in the *aba3* mutant, demonstrating that local ABA production in guard cells is essential for this response (Batool *et al*., 2018). Guard cells are not connected via plasmodesmata to the pavement or mesophyll cells and thus rely on the direct import of sulfate from the apoplast for assimilation (Fig. 2). Indeed, sulfate uptake and assimilation genes are highly expressed in Arabidopsis guard cells (Yoshimoto *et al*., 2002; Bauer *et al*., 2013), and guard cells possess photosynthetically active chloroplasts that can support sulfate reduction to sulfide (Santelia & Lawson, 2016). Furthermore, cysteine and sulfate can trigger stomata closure in the *abcg40* mutant, in which guard cells cannot import ABA from the apoplast (Kuromori *et al*., 2018), strongly supporting the idea that early sensing of sulfate acting as an early long‐distance drought signal occurs predominantly, if not exclusively, in guard cells. CLE25, the other xylem‐transported long‐distance water‐limitation signal, also triggers guard cell‐autonomous ABA biosynthesis by docking to specific receptors exposed at the guard cell plasmamembrane (Takahashi *et al*., 2018). Although CLE25 may regulate sulfate transport in roots (Sun *et al*., 2025), sulfate and CLE25 seem to act in parallel in guard cells to reinforce stomatal closure under drought. These findings emphasize the guard cells as essential hubs for sensing signals of soil water limitation.

The relevance of pCSC to stress‐induced stomatal closure is not limited to sensing long‐distance signals of soil water limitation. The pCSC is also essential for rapid high‐light stress‐induced stomatal closure (Sun *et al*., 2025). This local response is triggered by the oxylipin 12‐oxo‐phytodienoic acid (OPDA), a precursor to jasmonic acid biosynthesis that also has its own signaling function. OPDA binds to the *peptidyl prolyl isomerase* cyclophilin 20‐3 (CYP20‐3), which promotes formation of the pCSC (Fig. 3; Park *et al*., 2013). Together with the pCSC and 2‐Cys‐peroxiredoxins A/B, CYP20‐3 forms the redox‐sensitive COPS module that is critical for the high‐light stress‐induced transcriptional acclimation response (Mueller *et al*., 2017). Current evidence indicates that OPDA‐triggered pCSC assembly is directly involved in rapid stomatal closure, whereas the COPS module appears to act in longer‐term adjustment of gene expression. Remarkably, OPDA also accumulates in the leaves upon drought stress, although jasmonic acid is not affected by this stress (Savchenko *et al*., 2014), implying a substantial role for the COPS module in the early water limitation response. Taken together, these findings suggest that OPDA‐dependent pCSC activation provides an early trigger for stomatal closure, while the COPS network supports redox and transcriptional adjustments during prolonged environmental stress.

## IV. Cytosolic sulfide controls various stress responses, including stomata closure

Hydrogen sulfide (H_2_S) is a toxic gas that strongly inhibits mitochondrial cytochrome c oxidase; sulfide is nowadays accepted as a signaling molecule in animals and plants. The primary source of sulfide production in plants is the assimilatory sulfate reduction pathway in the chloroplast, which is tightly regulated to mitigate sulfide toxicity. However, other sulfide sources in plant cells are cysteine‐degrading activities like β‐cyanoalanine synthase, d‐cysteine desulfhydrases, and l‐cysteine desulfhydrase (DES), of which the cytosolic DES1 is best characterized (reviewed in Liu & Xue, 2021).

Sulfide regulates diverse developmental processes, including root development, leaf senescence, and fruit ripening, and is involved in responses to several abiotic stresses, including heat, salinity, and drought (Aroca *et al*., 2025). In addition to being sensed by CSC, sulfide can affect metabolism by binding to metal cofactors of metalloproteins or reacting with the thiol groups of cysteine residues in proteins (Liu & Xue, 2021; Aroca *et al*., 2025). The latter post‐translational modification, termed persulfidation, is considered the dominant sulfide signaling mechanism.

A quantitative proteomics approach revealed that drought stress can trigger persulfidation of 887 proteins in the leaves of Arabidopsis plants, which was accompanied by increased levels of cysteine and its downstream products GSH and sulfide (Jurado‐Flores *et al*., 2023). Indeed, fumigation of leaves with sulfide causes stomata closure. Still, it is hard to determine whether the applied sulfide acts directly on proteins or is incorporated into cysteine to trigger ABA production. That cytosolic sulfide can directly modulate stomata closure is supported by the following findings: The sulfide‐releasing enzyme DES1 is transcriptionally induced by ABA, and stomata of *des1* mutants are insensitive to ABA, suggesting that ABA‐induced DES1 is critical for stomatal closure (Scuffi *et al*., 2014). Furthermore, several vital regulators of ABA‐induced stomatal closure have been shown to be persulfidated, including the ABA‐responsive transcription factor ABI4 (Zhou *et al*., 2021), the sensor kinase OPEN STOMATA1 (OST1; Chen *et al*., 2021), and the OST1 substrates: SLOW ANION CHANNEL 1 (SLAC1; Wang *et al*., 2016) and RESPIRATORY BURST OXIDASE HOMOLOG D (RBOHD; Shen *et al*., 2020; reviewed in Liu & Xue, 2021). Since the former are known transducers of ABA‐mediated stomata closure and their activities are subject to rapid post‐translational modification, sulfide might well be a significant component of the stomatal aperture tuning system (Fig. 3). It is noteworthy that OST1 activity is controlled by several post‐translational modifications, including CLE5‐mediated phosphorylation and sulfide‐mediated persulfidation (Chen *et al*., 2021; Shimotohno *et al*., 2025), demonstrating its role as an integration point for different signals during stomatal closure. OST1, SLAC1, and RBOHD, which are the targets of SAL1‐PAP retrograde signaling‐activated calcium‐dependent kinases (Tee *et al*., 2025), are also essential for sulfate‐induced stomatal closure, but this does not necessarily mean that sulfate‐induced stomatal closure is upstream of sulfide‐mediated stomatal closure which requires ABA perception and ABA synthesis pathway (Malcheska *et al*., 2017; Batool *et al*., 2018; Rajab *et al*., 2019).

## V. Conclusions and outlook

Several lines of evidence reveal the roles of xylem‐mediated sulfate transport and cytosolic sulfide production in stomatal closure during drought stress. Very recent research has demonstrated that the assembly of the plastid‐localized CSC in the guard cells triggers stomatal closure in response to soil‐drying signals, sulfate, and CLE25, and the high‐light signal OPDA. Since CSC formation in the chloroplast of guard cells is required and sufficient for induction of ABA production and stomatal closure upon both stresses, guard cell‐autonomous *de novo* ABA biosynthesis appears to be more critical for early stress‐induced stomatal closure than previously anticipated.

Despite these progresses, several important questions remain unresolved. It is currently unclear how pCSC‐triggered cysteine formation in chloroplasts promotes ABA biosynthesis in the cytosol. In particular, the identity and regulation of cysteine export from chloroplasts require further investigation. The mechanism by which cysteine influences transcription of *NCED3*, encoding the rate‐limiting enzyme in ABA biosynthesis, is also unresolved. Future studies that combine cell‐type‐specific transcriptomics with targeted manipulation of cysteine levels in guard cells could dissect whether this regulation is direct or involves additional signaling steps. In parallel to this ABA biosynthesis‐inducing mechanism, degradation of cysteine by cytosolic DES1 might also contribute to cysteine‐induced stomatal closure by modifying numerous transducers of the ABA perception machinery via persulfidation. Identifying these protein targets and assessing their relative importance will require systematic analysis using mutants impaired in sulfide production together with proteomic approaches in guard cells. Incorporating sulfate transport, cysteine synthesis, and sulfide signaling into existing models of stomatal regulation will be essential for a comprehensive understanding of how plants coordinate metabolic status with environmental stresses by fine‐tuning hormone‐driven processes.

## Competing interests

None declared.

## Author contributions

MW, RH and S‐KS discussed the conceptual framework of the manuscript. MW wrote the manuscript with input from RH and S‐KS. S‐KS generated the figures with input from MW.

## Disclaimer

The New Phytologist Foundation remains neutral with regard to jurisdictional claims in maps and in any institutional affiliations.
